# Host plants and pollination regions for the long‐distance migratory noctuid moth, *Hadula trifolii* Hufnagel in China

**DOI:** 10.1002/ece3.8819

**Published:** 2022-04-11

**Authors:** Limei He, Yongqiang Liu, Jianglong Guo, Hong Chang, Kongming Wu

**Affiliations:** ^1^ 12661 Institute of Urban Agriculture Chinese Academy of Agricultural Sciences Chengdu China; ^2^ 12661 State Key Laboratory for Biology of Plant Diseases and Insect Pests Institute of Plant Protection Chinese Academy of Agricultural Sciences Beijing China; ^3^ Key Laboratory of Integrated Pest Management on Crops in Northern Region of North China Ministry of Agriculture and Rural Affairs IPM Center of Hebei Province Plant Protection Institute Hebei Academy of Agricultural and Forestry Sciences Baoding China; ^4^ Guangdong Provincial Key Laboratory of High Technology for Plant Protection Plant Protection Research Institute Guangdong Academy of Agricultural Sciences Guangzhou China

**Keywords:** *Hadula trifolii*, insect migration, pollen identification, pollinator

## Abstract

Nocturnal moths are important pollinators of plants. The clover cutworm, *Hadula trifolii*, is a long‐distance migratory nocturnal moth. Although the larvae of *H*. *trifolii* are polyphagous pests of many cultivated crops in Asia and Europe, the plant species pollinated by the adult are unclear. Pollen species that were attached to individual migrating moths of *H*. *trifolii* were identified based on pollen morphology and DNA to determine their host plants, geographic origin, and pollination areas. The moths were collected on their seasonal migration pathway at a small island, namely Beihuang, in the center of the Bohai Sea of China during 2014 to 2018. Pollen was detected on 28.60% of the female moths and 29.02% of the male, mainly on the proboscis, rarely on compound eyes and antennae. At least 92 species of pollen from 42 plant families, mainly from Asteraceae, Amaranthaceae, and Pinaceae, distributed throughout China were found on the test moths. Migratory *H*. *trifolii* moths visited herbaceous plants more than woody plants. Pollen of *Macadamina integrifolia* or *M*. *tetraphylla* was found on moths early in the migratory season. These two species are distributed in Guangdong, Yunnan, and Taiwan provinces in China, indicating that migratory moths probably traveled about 2000 km from southern China to the Beihuang Island in northern China. Here, by identifying plant species using pollen, we gained a better understanding of the interactions between *H*. *trifolii* moths and a wide range of host plants in China. This work provides valuable and unique information on the geographical origin and pollination regions for *H*. *trifolii* moths.

## Graphical abstract

Nocturnal moths are important pollinators of plants. The *Hadula trifolii* moths collected on Beihuang Island in the spring probably traveled about 2000 km from southern China.

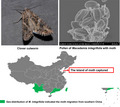

## 1 INTRODUCTION

Latitudinal migrations of billions of animals on land or through the water or air lead to great seasonal exchanges of biomass and nutrients across the Earth (Alerstam & Bäckman, 2018; Chapman et al., 2015; Dingle & Drake, 2007; Guo et al., 2020; Hu et al., 2016). The migration of insects, the most species‐rich and abundant group of macroscopic organisms on the planet, is linked to numerous ecosystem services including pollination, biological invasion, niche competition, outbreaks of agricultural and forestry pests, over large areas (Hendrix et al., 1987; Song et al., 2021; Wäckers et al., 2007; Weiner et al., 2014; Zhang et al., 2022). Tracking the movement of insects in their natural habitat is thus essential for understanding their basic biology, demography, ethology, and ecological function.

Moths are the major nocturnal pollinators of plants (Devoto et al., 2011; Lecroy et al., 2013). At least 289 species of plants from 75 families are partially or exclusively pollinated by moths belonging to 21 families (Macgregor et al., 2015). Moths visit flowers and feed on nectar and/or pollen to meet energy needs for flight and nutritional requirements for reproduction. As a result of this visitation and feeding activity, moths pick up pollen, which can be used to identify the plant species. Thus, pollen is an outstanding natural marker for mark–capture studies of insect migration and their host plants (Bryant et al., 1991; Chang et al., 2018; Guo et al., 2018; Hagler & Jackson, 2001; Lingren et al., 1993; Liu et al., 2016; Liu, Fu, et al., 2017).

Pollen identification is remarkably useful to study the movement of insects and insect–plant interactions for three reasons (Hagler & Jackson, 2001). Firstly, the hard outer wall of pollen grains is composed of sporopollenin, one of the most durable protein materials (Hagler & Jackson, 2001; Mackenzie et al., 2015). Secondly, the distinctive morphological characteristics of pollen grains enable it to be identified to the genus level (Guo et al., 2018; Hesse et al., 2009). Thirdly, the distribution and flowering period of most plants are also well known, which helps to determine the geographic origin of collected insects (Chang et al., 2018; Hendrix et al., 1987; Liu et al., 2016; Liu, Fu, et al., 2017).

Pollen grains can be identified to the genus or even the species level using light microscopy (LM), scanning electron microscopy (SEM), and DNA metabarcoding. Light microscopy for pollen identification is constrained by low resolution, and preparation methods often generate confusing contaminants such as insect lipids and chitins (Hagler & Jackson, 2001; Turnock et al., 1978). Although SEM allows direct observation of the attached pollen grains with more detail and higher resolution than LM, it is costlier and more time‐consuming (Bryant et al., 1991; Hagler & Jackson, 2001; Turnock et al., 1978). Since the initial introduction of DNA metabarcoding, DNA‐assisted identification of pollen grains has become common for identifying biological species, insect feeding preferences, and host plant distribution, and the origin of migratory insects (Chang et al., 2018; Galliot et al., 2017; Hawkins et al., 2015; Hebert et al., 2003; Jackson & Gahr, 2019; Liu et al., 2016; Liu, Fu, et al., 2017).

**FIGURE 1:**
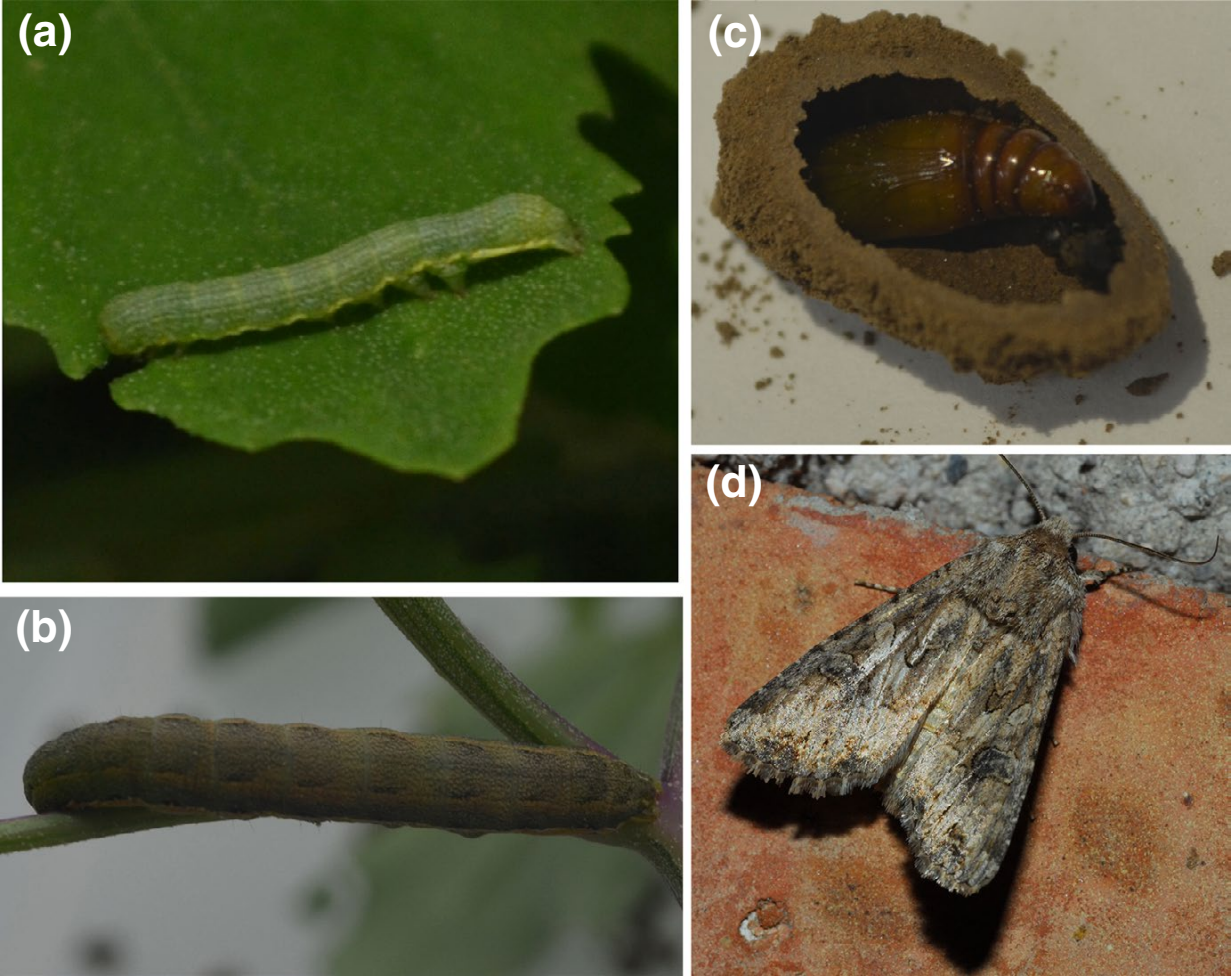
Representative images of *Hadula trifolii* (a: young larva; b: older larva; c: pupa chamber and pupa; d: adult). All images were taken by the author of this article with Nikon D5100 (a–c) and D200 (d)

In this study, we used light microscopy, scanning electron microscopy, and DNA metabarcoding to identify the pollen species attached to migratory moths of the clover cutworm, *Hadula trifolii* Hufnagel (synonyms: the nutmeg; *Apamea inquieta*; *Discestra trifolii*; *Hadena albifusa*; *Scotogramma trifolii*) (Lepidoptera: Noctuidae) (Figure 1), an agricultural pest in northern China and a common species in the community of insects that migrate across the Bohai Sea (Fu, 2015; Zhang et al., 2010; Zhao et al., 1992; Zhou et al., 2020). It is globally distributed in both subtropical and temperate regions including Asia, Europe, North Africa, and North America (Federici, 1978; He, 1997; Zhang & Yu, 2021). Its larvae are a serious agricultural threat because they feed on more than 20 cultivated crop species, including potato, beet, cabbage, sunflower, wheat, corn, cotton, apple, melon, and legumes (Cass, 1959; Ren et al., 2006; Yu & Bao, 1996; Zhang & Yu, 2021; Zhao et al., 1992). In addition, *H*. *trifolii* is a long‐distance migratory insect (Zhang et al., 2010). They migrate toward the north in prevailing southerly winds during late spring (May) and early summer (June and July) and return to the south in prevailing northerly winds during late summer and early autumn (August to October) (He et al., 2018). However, the geographical origin and migratory paths of these migratory populations are still unknown. To better estimate the risk of these insect pests to agriculture, we need to understand the origins, ranges, and food sources.

The food source is also likely to affect reproductive and migratory fitness. Migratory noctuid moths are often contaminated with pollen (Chang et al., 2018; Hendrix et al., 1987; Hendrix & Showers, 1992; Liu et al., 2016; Liu, Fu, et al., 2017; Zhou et al., 2019), and adult feeding (pollen or nectar) can significantly increase the longevity, flight, and reproductive capability of lepidopteran insects (Gilbert, 1972; He et al., 2021; Liu, Zhu, et al., 2017; Wäckers et al., 2007; Wu & Guo, 1997). However, it is still unclear which plant species are hosts for migratory *H*. *trifolii* moths, where the moths originate and the areas in which the moths pollinate flowers.

In the present study, we defined the host plants, geographic origin, and pollination area of *H*. *trifolii* moths by identifying and quantifying the pollen grains that adhered to various parts of their heads during their long‐distance migration. This work validates the application of DNA‐based palynology in aerobiological and ecological study of lepidopterans. Our results provide evidences that *H*. *trifolii* moths are important nocturnal pollinators for a diverse range of plants in China and enrich our understanding of the interactions between agricultural pests and plants.

## 2 MATERIALS AND METHODS

### 2.1 Collection of migratory moths

Migratory moths of *H*. *trifolii* were captured using vertically aimed searchlight traps during 2014–2018 at Beihuang Island (Bohai Strait, China; 38°24′N, 120°55′E) (Feng et al., 2003; He et al., 2018). Twenty moths (♀:♂ =1:1; or all individuals if the total captured was <20) were removed from the nylon net capture bag every morning and placed singly into 2 ml tubes and stored in a −20°C freezer until microscopic inspection.

### 2.2 Pollen examination and scanning electron microscopy (SEM) preparation

Pollen is usually observed on the proboscis, antennae, compound eyes, and legs of moths (Bryant et al., 1991; Liu et al., 2016). To clear the presence of pollen, the heads of adult *H*. *trifolii* were excised and examined with an Olympus SZX16 stereomicroscope. To prevent contamination, we washed the microscope slide under sample and all forceps with ethanol before examining each new sample (Liu et al., 2016; Liu, Fu, et al., 2017). Pollen grains found on the head (i.e., proboscis, antennae, and eyes) were placed on double‐sided sticky tape on aluminum stubs, sputter‐coated with gold in a sputter coater and imaged with a Hitachi S‐4800 or SU8010 cold field emission SEM (Hitachi High‐Technologies Co.).

### 2.3 Pollen lysis and single pollen PCR

A single pollen grain was picked from the aluminum stubs using a plastic pipette tip (micropipette puller; Sutter Instruments) with an Olympus SZX16 stereomicroscope and placed into 5 μl of lysis solution (0.1 M NaOH, plus 2% Tween‐20; Beijing Chemical Reagent Co. Ltd) in separate PCR tubes (Chang et al., 2018; Chen et al., 2008; Liu et al., 2016). DNA was extracted from single pollen grains and preserved as reported previously (Chang et al., 2018; Liu et al., 2016) and then used for PCR of a partial region of the chloroplast gene *rbcL* and ITS using the respective primer pairs: *rbcla* forward (5′‐ATGTCACCACAAACAGAAAC‐3′) and reverse (5′‐TCGCATGTACCTGCAGTAGC‐3′)/*rbclb* forward (5′‐ATGTCACCACAAACAGAAAC‐3′) and reverse (5′‐GAAACGGTCTCTCCAACGCAT‐3′); ITS forward (5′‐GACTCTCGGCAACGGATATC‐3′)/ITS reverse (5′‐TCCTCCGCTTATTGATATGC‐3′) (Chang et al., 2018). The PCR mixture and thermocycling conditions of Chang et al. (2018) were used with the GeneAmp PCR System 9700 thermocycler (Applied Biosystems).

The separation and purification methods of PCR amplicons were the same as those of Chang et al. (2018). The purified products were subcloned into pEASY‐T3 Cloning Vector (TransGen Biotech). The inserts were then sequenced with standard M13 primers (Shanghai Sangon), and Sanger sequencing of pollen was done by the Taihe Biotechnology Co., Ltd.

### 2.4 Pollen identification and characteristics of pollen source plants

Morphological features of pollen grains were examined and species identified using *Pollen Terminology* (Hesse et al., 2009), *Pollen Flora of China Woody Plants by SEM* (Li et al., 2010), *Pollen Morphology of Inner Mongolian Plants* (Wan et al., 2020), and the palynological database PalDat 3.3 (available online: https://www.paldat.org/) and literature (Bryant et al., 1991; Chang et al., 2018; Guo et al., 2018; Lingren et al., 1993; Liu et al., 2016; Liu, Fu, et al., 2017; Zhou et al., 2019). DNA sequences of pollen grains were used to identify family, genus, and/or species based on a similarity search of the GenBank database (Altschul et al., 1990) using the Basic Local Alignment Search Tool (BLAST, available online: https://blast.ncbi.nlm.nih.gov/Blast.cgi). Pollen grain types that could be identified and classified to family, genus, or even species level were used to identify the source plants of pollen based on distribution data of plants in China from the database iPlant (available online: http://www.iplant.cn/).

### 2.5 Data analyses

Differences in taxa and number of *H*. *trifolii* moths with adhering pollen per season or per year (frequency) during different migratory seasons were analyzed using a one‐way analysis of variance (ANOVA), of proportional data that were first arcsine square‐root‐transformed to meet assumptions of normality and heteroscedasticity. Tukey's honestly significant difference (HSD) was used as a post hoc test. The annual mean frequencies of pollen deposits on male and female moths were compared for differences using Student's *t* test. Differences in annual percentages of pollen on male and female moths and the characteristics of pollen source plants were all compared using a chi‐squared test. All statistical analyses were done in SPSS 20.0 (IBM), except for the log rank test, which was done in GraphPad Prism 8 (GraphPad Software Inc.).

## 3 RESULTS

### 3.1 Plant hosts deduced from pollen

**FIGURE 2:**
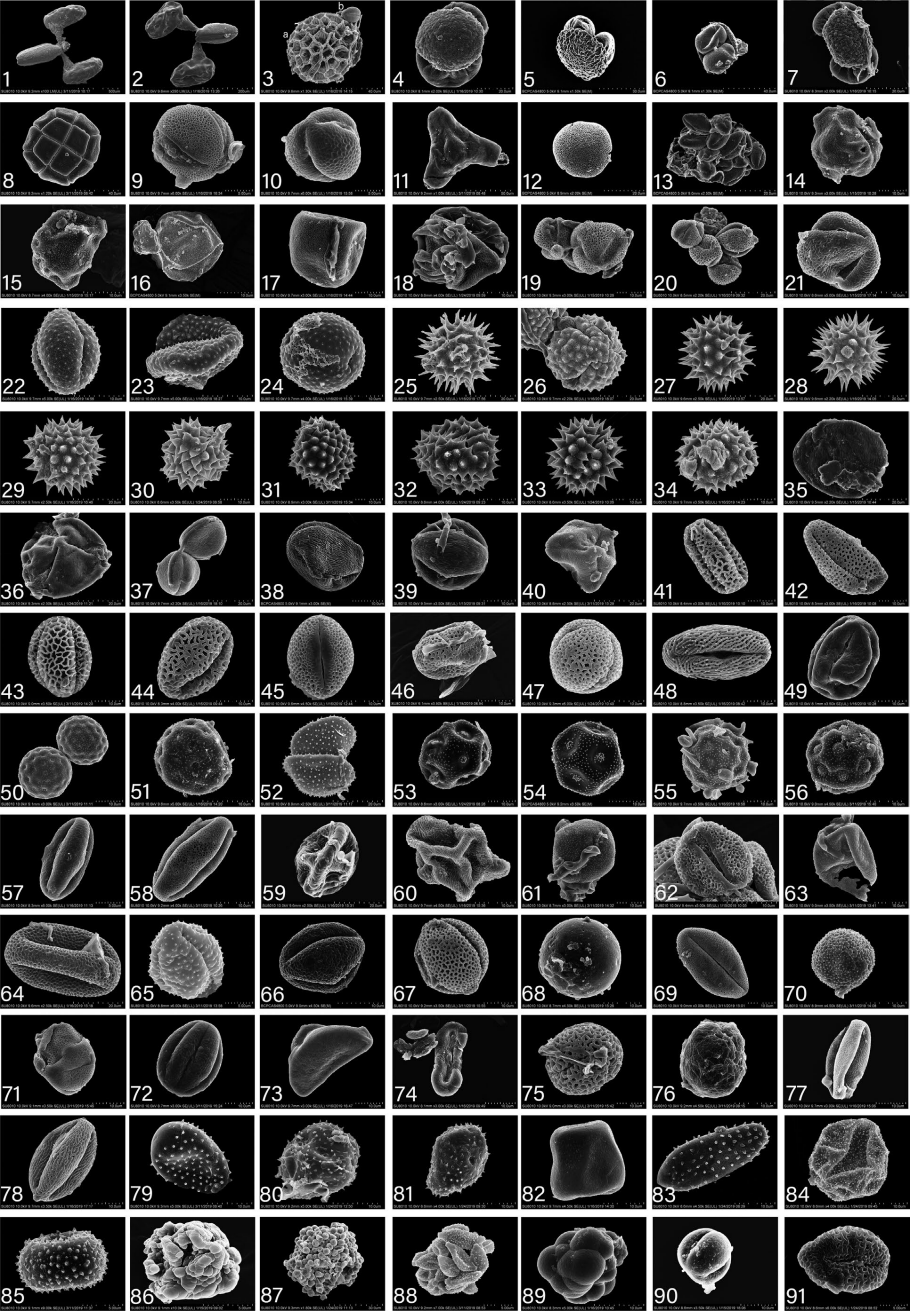
Scanning electron micrographs (SEM) of the pollen grains found adhering to *Hadula trifolii* moths. Species information for all pollen is given in the Table 1. Scale bars at bottom of images—1: 500 μm; 2: 200 μm; 3, 6, 8: 40 μm; 4, 7, 12, 13, 20, 25–29, 35–37, 40, 52, 59,64: 20 μm; 5,87: 30 μm; 9, 10, 57, 65, 85, 86, 88, 91: 5 μm; 11: 50 μm; 14–19, 21–24, 30–34, 38, 39, 41–51, 53–56, 58, 60–63, 66–84, 89–90: 10 μm

During the study, 1985 moths of *H*. *trifolii* were collected; 27% had pollen grains on the proboscis, 2.32% had pollen on the antennae, and 0.6% on compound eyes. For most individuals that had pollen adhering to the body (i.e., 89.16%), the pollen was from one species; the remainder had pollen from two or three species. Ninety‐two pollen species from at least 42 families were discovered on the moths (Table 1, Figure 2, Text S1). Using both DNA sequences and pollen morphology, we were able to identify 17 samples to the species level: *Pinus densiflora* Sieb. et Zucc., *Pinus bungeana* Zucc. ex Endl, *Pinus taiwanensis* Hayata, *Amorpha fruticosa* L., *Lablab purpureus* (L.) Sweet, *Oryza sativa* L., *Zea mays* L., *Helianthus annuus* L., *Cosmos bipinnatus* Cavanilles, *Ambrosia artemisiifolia* L., *Flueggea virosa* (Roxb. ex Willd.) Voigt, *Alniphyllum fortunei* (Hemsl.) Makino, *Ailanthus altissima* (Mill.) Swingle, *Melia azedarach* L., *Chenopodium album* L., *Adenophora trachelioides* Maxim., *Stellera chamaejasme* L. For 50 samples, pollen was identified to the genus, and 47 genera were found: *Cynanchum* L., *Vincetoxicum* Wolf, *Limonium* Mill., *Pinus* L., *Albizia* Durazz., *Oenothera* L., *Tilia* L., *Macadamia* F. Muell., *Corylus* L., *Carpinus* L., *Salix* L., *Deutzia* Thunb., *Artemisia* L., *Cirsium* Mill., *Bidens* L., *Aster* L., *Zinnia* L., *Rosa* L., *Prunus* L., *Spiraea* L., *Syringa* L., *Fraxinus* L., *Jasminum* L., *Citrus* L., *Chenopodium* L., *Dianthus* L., *Stellaria* L., *Alisma* L., *Castanea* Mill., *Vitis* L., *Cornus* L., *Nicotiana* L., *Solanum* L., *Platanus* L., *Polygonum* L., *Fagopyrum* Mill., *Papaver* L., *Embelia* Burm. F., *Brassica* L., *Juniperus* L., *Asparagus* L., *Elsholtzia* Willd., *Clinopodium* L., *Allium* L., *Ulmus* L., *Camellia* L., and *Lomatogonium* A. Braun. Overall, 78 type of pollen from 42 families were identified, and type for 14 pollen grains were unidentified. Thus, the identification success rate using a combination of pollen morphology and DNA sequences was 17.91% to species and 41.80% to genus. Using DNA sequences only, the rate was 5.97% to species and 55.22% to genus, and pollen morphology only, the rate was 4.48% to species and 31.30% to genus.

**TABLE 1:** Pollen grains carried by *Hadula trifolii* moths and type identified by molecular and morphological analysis and the geographic distribution of the pollen source plants

Pollen grain type	Identified plants	Morphology‐based identification	Molecular identification	Geographic distribution in China
1	*Cynanchum* spp.	Asclepiadaceae	Sister to *Cynanchum thesioides*/*Cynanchum acidum*/*Cynanchum wilfordii*	Southwest, northwest, and northeast China
2	*Vincetoxicum* spp.	*Vincetoxicum* spp.	Unidentifiable	Southwest, northwest, and northeast China
3a	*Limonium* spp.	*Limonium* spp.	Unidentifiable	Northwest, northeast, north of China and coastal areas, Xinjiang, Tibet, Henan
4	*Pinus densiflora*	*Pinus densiflora*	Sister to *Pinus sylvestris*/*Pinus yunnanensis*/*Pinus densiflora*	Northeast China
5	*Pinus bungeana*	*Pinus bungeana*	Sister to *Pinus bungeana*/*Pinus squamata*	Beijing, Shanxi, Henan, Shaanxi, Gansu, Sichuan, Hubei, Hubei, Jiangsu
6	*Pinus taiwanensis*	*Pinus taiwanensis*	Sister to *Pinus taiwanensis*	Taiwan, Fujian, Zhejiang, Anhui, Jiangxi, Hunan, Hubei, Henan
7	*Pinus* spp.	*Pinus* spp.	Sister to *Pinus* spp.	The nationwide distribution
8	*Albizia* spp.	*Albizia* spp.	Unidentifiable	South, southwest and southeast China
9	*Amorpha fruticosa*	*Amorpha fruticosa*	Sister to *Amorpha glabra*/*Amorpha fruticosa*/*Amorpha nana*	northeast, north, northwest of China and Shangdong, Anhui, Jiangsu, Henan, Hubei, Guangxi, Sichuan
10	*Lablab purpureus*	Fabaceae	Sister to *Lablab purpureus*	Throughout China
11	*Oenothera* spp.	Onagraceae	Sister to *Oenothera* spp.	Throughout China
12	*Tilia* spp.	*Tilia* spp.	Unidentifiable	South of the Yellow River Basin
13	*Macadamia integrifolia/Macadamia tetraphylla*	Proteaceae	Sister to *Macadamia integrifolia*/*Macadamia tetraphylla*	Yunnan, Guangdong, Taiwan
14	*Corylus* spp.	*Corylus* spp.	Sister to *Corylus* spp.	From southwest to northeast China
15	*Carpinus* spp.	*Carpinus* spp.	Sister to *Carpinus* spp.	From southwest to northeast China
16	*Oryza Sativa*	Poaceae	Sister to *Oryza sativa*/*Oryza coarctata*	Throughout China
17	*Zea mays*	Poaceae	Sister to *Zea mays*/*Tripsacum dactyloides*	Throughout China
18	Poaceae	Poaceae	Unidentifiable	Throughout China
19	*Salix* spp.	*Salix* spp.	Sister to *Salix* spp.	Throughout China
20	*Salix* spp.	*Salix* spp.	Unidentifiable	Throughout China
21	*Deutzia* spp.	*Deutzia* spp.	Sister to *Deutzia* spp.	Throughout China
22	*Artemisia* spp.	*Artemisia* spp.	Sister to *Artemisia japonica*/*Artemisia hallaisanensis*/*Artemisia capillaris*	Throughout China
23	*Artemisia* spp.	*Artemisia* spp.	Sister to *Artemisia princeps*/*Artemisia vulgaris*/*Artemisia lavandulifolia*	Throughout China
24	*Artemisia* spp.	*Artemisia* spp.	Sister to *Artemisia stolonifera*/*Artemisia selengensis*/*Artemisia princeps*	Throughout China
25	*Helianthus annuus*	*Helianthus annuus*	Sister to *Helianthus annuus*/*Helianthus argophyllus*/*Helianthus debilis*	Throughout China
26	*Cirsium* spp.	*Cirsium* spp.	Unidentifiable	Throughout China
27	*Cosmos bipinnatus*	*Cosmos bipinnatus*	Unidentifiable	Throughout China
28	*Bidens* spp.	*Bidens* spp.	Unidentifiable	Throughout China
29	*Aster* spp.	*Aster* spp.	Unidentifiable	Throughout China
30	*Zinnia* spp.	*Zinnia* spp.	Unidentifiable	Throughout China
31	*Ambrosia artemisiifolia*	*Ambrosia artemisiifolia*	Unidentifiable	Liaoning, Jilin, Heilongjiang, Hebei, Shandong, Jiangsu, Jiangxi, Anhui, Hunan, Hubei
32	Asteraceae	Asteraceae	Unidentifiable	Throughout China
33	Asteraceae	Asteraceae	Unidentifiable	Throughout China
34	Asteraceae (*Inula* spp.)	Asteraceae (*Inula* spp.)	Unidentifiable	Throughout China
35	*Rosa* spp.	*Rosa* spp.	Sister to *Rosa* spp.	Throughout China
36	*Prunus* spp.	*Prunus* spp.	Sister to *Prunus* spp.	Throughout China
37	*Spiraea* spp.	*Spiraea* spp.	Unidentifiable	Throughout China
38	Rosaceae	Rosaceae	Unidentifiable	Throughout China
39	Rosaceae	Rosaceae	Unidentifiable	Throughout China
40	Rosaceae	Rosaceae	Unidentifiable	Throughout China
41	*Syringa* spp.	Oleaceae	Sister to *Syringa* spp.	Throughout China
42	*Fraxinus* spp.	Oleaceae	Sister to *Fraxinus* spp.	Throughout China
43	*Jasminum* spp.	*Jasminum* spp.	Unidentifiable	Throughout China
44	Oleaceae	Oleaceae	Unidentifiable	Throughout China
45	*Flueggea virosa*	*Flueggea virosa*	Sister to *Flueggea neowawraea*/*Flueggea virosa*	Eastern, southern and southwestern China
46	*Citrus* spp.	*Citrus* spp.	Sister to *Citrus* spp.	Shaanxi, Gansu and the area south of the Qinling Mountains
47	*Alniphyllum fortunei*	*Alniphyllum fortunei*	Sister to *Alniphyllum* spp.	Southern China
48	*Ailanthus altissima*	*Ailanthus altissima*	Sister to *Ailanthus altissima*	Throughout China
49	*Melia azedarach*	*Melia azedarach*	Sister to *Melia azedarach*	South of the Yellow River
50	*Chenopodium album*	*Chenopodium album*	Sister to *Oxybasis glauca*/*Chenopodium ficifolium*/*Chenopodium album*	Throughout China
51	*Chenopodium* spp.	*Chenopodium* spp.	Unidentifiable	Throughout China
52	*Adenophora trachelioides*	*Adenophora trachelioides*	Sister to *Adenophora trachelioides*/*Adenophora tetraphylla*	Beijing, Liaoning, Hebei, Shandong, Jiangsu, Zhejiang, Anhui, Shanxi
53	*Dianthus* spp.	*Dianthus* spp.	Sister to *Dianthus* spp.	Throughout China
54	*Stellaria* spp.	*Stellaria* spp.	Unidentifiable	Throughout China
55	Caryophyllaceae	Caryophyllaceae	Unidentifiable	Throughout China
56	*Alisma* spp.	*Alisma* spp.	Unidentifiable	Throughout China
57	*Castanea* spp.	*Castanea* spp.	Sister to *Castanea* spp.	Throughout China
58	*Vitis* spp.	*Vitis* spp.	Sister to *Vitis* spp.	Throughout China
59	*Cornus officinalis*/*Cornus chinensis*	*Cornus* spp.	Sister to *Cornus officinalis*/*Cornus eydeana*/*Cornus chinensis*	Guangdong, Sichuan, Guizhou, Yunnan, Shanxi, Shaanxi, Gansu, Shandong, Jiangsu, Zhejiang, Anhui, Jiangxi, Henan, Hunan, Hubei
60	*Nicotiana* spp.	*Nicotiana* spp.	Sister to *Nicotiana* spp.	Throughout China
61	*Solanum* spp.	*Solanum* spp.	Sister to *Solanum* spp.	Throughout China
62	*Platanus* spp.	*Platanus* spp.	Sister to *Platanus* spp.	Throughout China
63	*Polygonum* spp.	*Polygonum* spp.	Sister to *Polygonum* spp.	Throughout China
64	*Fagopyrum* spp.	*Fagopyrum* spp.	Unidentifiable	Throughout China
65	*Papaver* spp.	*Papaver* spp.	Unidentifiable	Throughout China
66	*Embelia* spp.	*Embelia* spp.	Sister to *Embelia* spp.	From southeast to southwest China
67	*Brassica* spp.	*Brassica* spp.	Sister to *Brassica* spp.	Throughout China
68	*Juniperus* spp.	Cupressaceae	Sister to *Juniperus* spp.	Throughout China
69	*Asparagus* spp.	*Asparagus* spp.	Unidentifiable	Throughout China
70	*Stellera chamaejasme*	*Stellera chamaejasme*	Unidentifiable	Northern provinces and southwestern China
71	*Elsholtzia* spp.	*Elsholtzia* spp.	Unidentifiable	Throughout China
72	*Clinopodium* spp.	*Clinopodium* spp.	Unidentifiable	Throughout China except Xinjiang
73	*Allium* spp.	*Allium* spp.	Unidentifiable	Northeast, north, northwest and southwest China
74	Apiaceae	Apiaceae	Unidentifiable	Throughout China
75	Araliaceae	Araliaceae	Unidentifiable	Throughout China
76	*Ulmus* spp.	*Ulmus* spp.	Sister to *Ulmus* spp.	Throughout China
77	*Camellia* spp.	*Camellia* spp.	Unidentifiable	Yunnan, Guangxi, Guangdong, Sichuan
78	*Lomatogonium* spp.	*Lomatogonium* spp.	Unidentifiable	Southwest China
3b, 79–91	Unknown			

### 3.2 Annual and seasonal differences in pollen‐carrying frequencies

For the 1985 moths of *H*. *trifolii* that were collected and observed for pollen grains, annual percentages of pollen‐bearing individuals differed among years (Table 2; *χ* ^2^ = 76.700, *df* = 4, *p* < .001). For the 572 pollen grains on the test moths, 42 families, 61 genera, and 17 species were identified (Table 2).

**TABLE 2:** Annual data for the percentage of *Hadula trifolii* moths with carrying pollen grains and the level of taxonomic resolution

No./%	2014	2015	2016	2017	2018	Total
No. adults examined	57	390	224	755	559	1985
No. adults with pollen	11	72	29	275	185	572
Adults with pollen (%)	19.30	18.46	12.95	36.42	33.09	28.82
No. plant families	8	10	7	31	28	42
No. plant genera	8	19	9	53	40	61
No. plant species	4	9	3	10	9	17
No. plant type	10	28	10	58	42	78

The relative percentage of male and female *H*. *trifolii* moths maintaining pollen demonstrated significant inter‐annual variation, with the highest level of pollen grain adhesion in 2017. On the whole, there were no distinguished sex‐related differences in the proportion of pollen grain adhesion on the moths among years (2014–2018), with 28.6% of female and 29.02% of male moths bearing pollens (Table 3).

**TABLE 3:** Results of chi‐squared test and Student's *t* test to compare frequencies of pollen grain attachment by year among male and female moths of *Hadula trifolii*

Year	Female	Male	*χ* ^2^	*df*	*p*
	No. (%) of moths with pollen				
2014	6 (23.08)	5 (16.13)	0.106	1	.745
2015	39 (17.57)	33 (19.64)	0.153	1	.696
2016	12 (11.11)	17 (14.66)	0.349	1	.555
2017	133 (37.15)	142 (35.77)	0.101	1	.750
2018	86 (34.26)	99 (32.14)	0.193	1	.660
			***t***	***df***	***p***
2014–2018	276 (28.60)	296 (29.02)	0.116	8	.911

**FIGURE 3:**
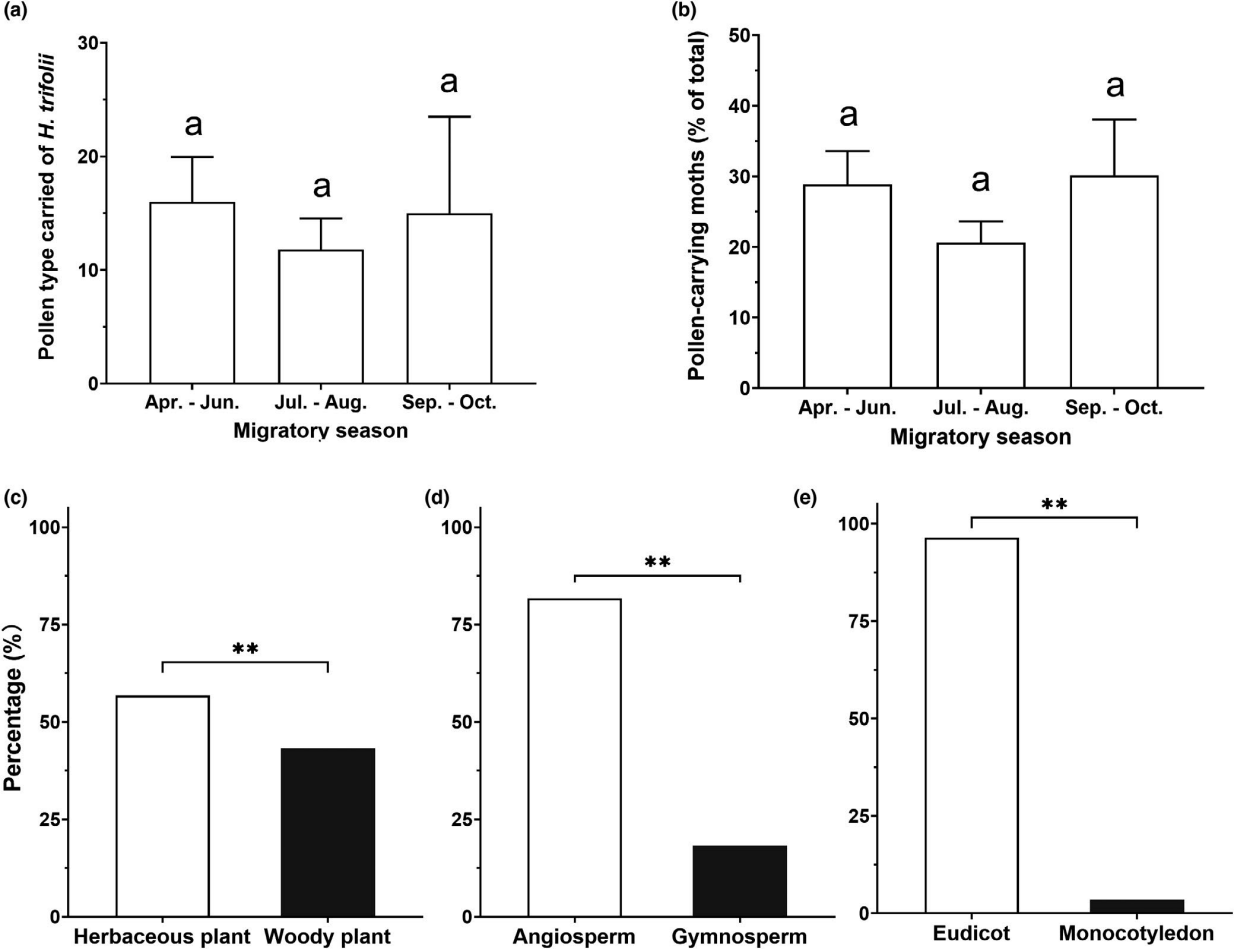
Number of type (a) and frequencies (b) of pollen grains and type of host plants (c, d, and e) represented by pollen grains attached to migratory individuals of *Hadula trifolii* in 2014–2018. Different letters above bars in panel a or b indicate a significant difference among means (*p* > .05, one‐way ANOVA followed by Tukey's HSD test), and double asterisks (**) in panels c–e indicates a significant difference between means (*p* < .01, chi‐squared test)

In addition, neither the number of identified pollen type, nor the frequency of pollen adherence on the moths differed among different migratory seasons during 2014 to 2018 (number of type: *F*_2, 10_ = 0.184, *p* = .835, Figure 3a; frequency: *F*_2, 10_ = 0.559, *p* = .589, Figure 3b).

### 3.3 Growth forms of pollen‐bearing host plants

The plant species identified for the adherent pollen grains represented a variety of growth forms: trees, shrubs, vines, and herbs. Herbaceous, angiosperm, and eudicot species were significantly more frequent than woody (*χ* ^2^ = 9.354, *df* = 1, *p* = .002; Figure 3c), gymnosperm (*χ* ^2^ = 204.968, *df* = 1, *p* < .001; Figure 3d), or monocotyledons (*χ* ^2^ = 358.164, *df* = 1, *p* < .001; Figure 3e).

### 3.4 Intra‐annual shifts in pollen taxa

Pollen grains from at least 28, 19, and 23 different plant families were recorded from *H*. *trifolii* migrants in the early (April and June), mid (July and August), and late (September and October) migratory season, respectively (Table 4). Among the pollen species in the early, mid, and late migratory season, 25.61%, 18.48%, and 13.73%, respectively, could not be identified. Pollen grains from Pinaceae (34.15%), Oleaceae (6.91%), and Rosaceae (5.69%) were most commonly recorded early in the migratory season. In the middle of the migratory season, the most frequent in from highest to lowest was Amaranthaceae (27.72%), Asteraceae (18.48%), and Campanulaceae (6.52%) compared with Asteraceae (34.8%), Amaranthaceae (18.63%), and Polygonaceae (5.88%) late in the season. Overall, pollen grains of Asteraceae (16.56%), Amaranthaceae (14.2%), and Pinaceae (13.56%) were found more frequently than any other family.

**TABLE 4:** Families and abundance of pollen grains attached to migrant *Hadula trifolii* moths captured in different periods of the migratory season on Beihuang Island in 2014–2018

Family	Early season (April–June)	Mid‐season (July–August)	Late season (September–October)	Migratory season (April–October)
Apocynaceae		3.80		1.10
Plumbaginaceae		0.54		0.16
Pinaceae	34.15	0.54	0.49	13.56
Fabaceae	2.85	1.63	2.45	2.37
Onagraceae	0.41	2.72		0.95
Malvaceae	0.41			0.16
Proteaceae	0.41			0.16
Betulaceae	3.25			1.26
Poaceae		1.09	1.96	0.95
Salicaceae	1.22			0.47
Hydrangeaceae	1.22			0.47
Asteraceae		18.48	34.80	16.56
Rosaceae	5.69	2.72	1.96	3.63
Oleaceae	6.91	0.54	0.98	3.15
Phyllanthaceae	1.63	7.61		2.84
Rutaceae	4.47			1.74
Styracaceae	1.22			0.47
Simaroubaceae	0.81			0.32
Meliaceae	1.63			0.63
Amaranthaceae	0.41	27.72	18.63	14.20
Campanulaceae		6.52	0.98	2.21
Caryophyllaceae	0.81	1.63	1.96	1.42
Alismataceae			0.49	0.16
Fagaceae	0.81			0.32
Vitaceae	0.41	1.09		0.47
Cornaceae	0.41		0.49	0.32
Solanaceae			1.47	0.47
Platanaceae	0.81			0.32
Polygonaceae	0.81	2.17	5.88	2.84
Papaveraceae			0.98	0.32
Primulaceae	0.41			0.16
Brassicaceae	0.41	0.54	1.96	0.95
Cupressaceae	1.22	0.54	1.47	1.10
Asparagaceae			0.49	0.16
Thymelaeaceae			0.98	0.32
Lamiaceae			1.47	0.47
Amaryllidaceae	0.41		2.94	1.10
Apiaceae	0.41			0.16
Araliaceae		0.54	0.98	0.47
Ulmaceae	0.81	1.09		0.63
Theaceae			0.49	0.16
Gentianaceae			1.96	0.63
Unknown	25.61	18.48	13.73	19.72

### 3.5 Area pollinated by *H. trifolii*

**FIGURE 4:**
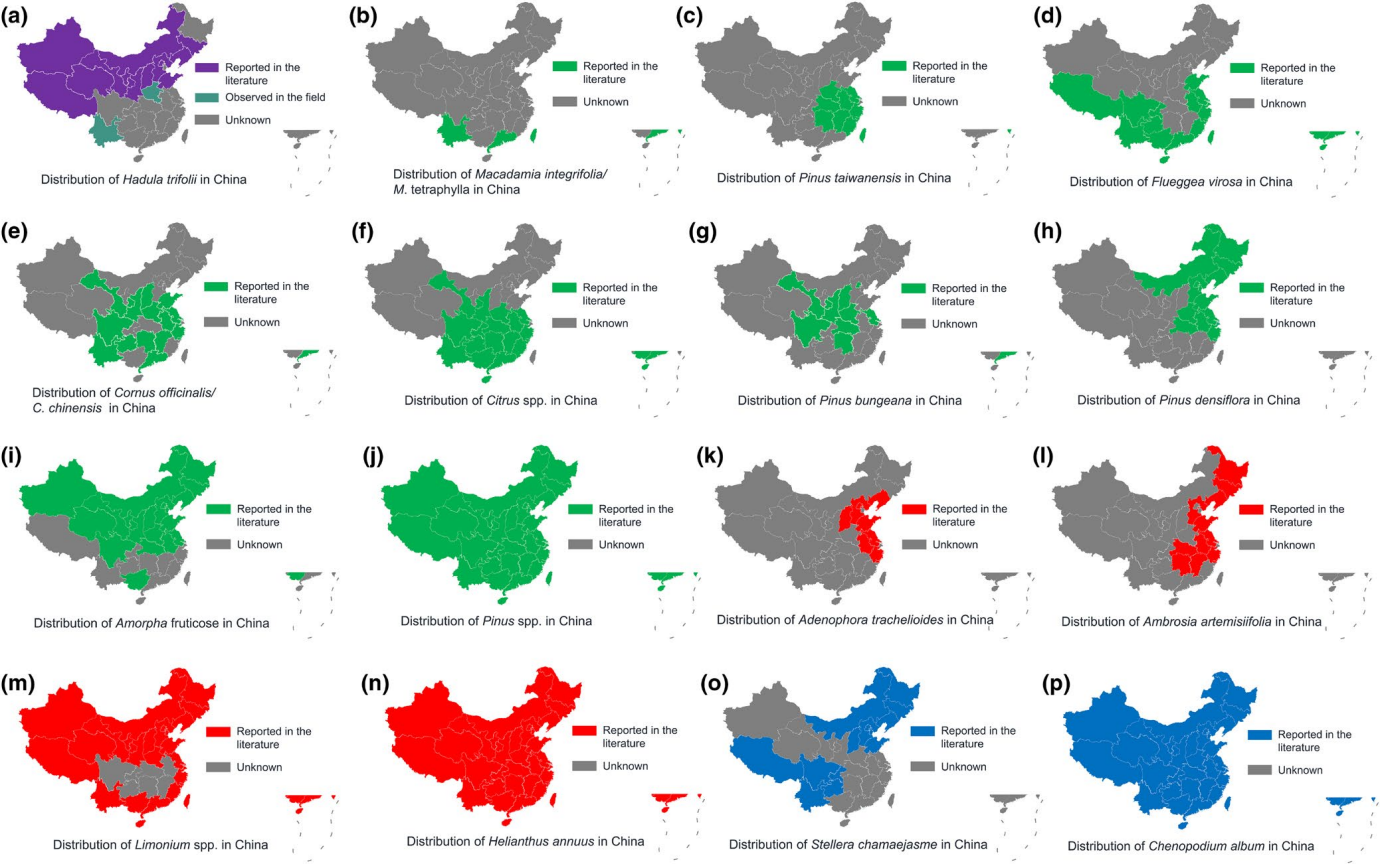
Distribution of *Hadula trifolii* (a) and plant species represented by pollen at different times during their migratory season (b–j: early season, April–June; k–n: mid‐season, July–August; o, p: late season, September–October) in China

In China, *H*. *trifolii* mainly occurs in Yunnan Province and in the northwestern and northern provinces (Figure 4a). Combining the pollen identification results and distribution information for plants in China, we found that the pollination area of *H*. *trifolii* moths extends to Shanghai in the east, Xinjiang in the west, Hainan in the south, and Heilongjiang in the north during the different migration times (Figure 4b–p).

## 4 DISCUSSION

In this study, we identified the range of host plants foraged by *H*. *trifolii* moths that migrated across the Bohai Sea by the integrated use of pollen morphology, DNA metabarcoding, and known geographical distribution of plant species identified. Our results indicated that migratory *H*. *trifolii* moths visited and obtained nectar and/or pollen from at least 92 plant species belonging to 42 families, which is in line with previous studies for other noctuid moths (Chang et al., 2018; Hendrix & Showers, 1992; Lingren et al., 1993; Liu et al., 2016; Liu, Fu, et al., 2017). Migratory *H*. *trifolii* moths may also visit gymnosperms (*Pinus* spp. and *Juniperus* spp.) and consume their nectar and/or pollen. This finding is similar to the results for *Agrotis ipsilon* Hufnagel (Liu et al., 2016), *Mythimna separata* Walker (Guo et al., 2018; Liu, Fu, et al., 2017), *Agrotis segetum* Denis and Schiffermaller (Chang et al., 2018) and *Helicoverpa armigera* Hübner (Zhou et al., 2019). Migratory *A*. *ipsilon* and *M*. *separata* moths (Liu et al., 2016; Liu, Fu, et al., 2017) visit woody plants more often than herbaceous plants. On the contrary, migratory *H*. *trifolii* moths evidently preferred herbaceous plants more than woody plants as does *A*. *segetum* (Chang et al., 2018). Different food sources can affect the structure and the temporal dynamics of an insect population (Wäckers et al., 2007). The quality and quantity of food sources can influence insect survival, development, flight, and reproduction (He et al., 2021; Wäckers et al., 2007), and the fitness of insects usually differs depending on its nectar and/or pollen sources (He et al., 2021; Liu, Zhu, et al., 2017). Of course, further study is needed to explore host‐plant feeding preferences of *H*. *trifolii* adults and assess the effects on population dynamics.

Although pollen identification can be used to determine the geographical origin of migratory insects, pollen can be picked up in regions far from where the pollen‐contaminated insects are captured (Hagler & Jackson, 2001). For example, Hendrix et al. (1987) found that male *Heliothis zea* Boddie moths captured in Arkansas probably originated from southern Texas, at least 750 km away, based on pollen from the proboscis or eye area. *Pseudaletia unipuncta* Haworth and *A*. *ipsilon* moths captured in Iowa and Missouri were contaminated with exotic pollen grains from species that only grow in southern Texas, which provided evidence that these two moths probably traveled 1300–1600 km to Iowa or Missouri from within Mexico (the state of Tamaulipas) (Hendrix & Showers, 1992). Previous studies have confirmed that *H*. *trifolii* is a long‐distance migratory pest (He et al., 2018; Zhang et al., 2010). We detected and identified pollen from *M*. *integrifolia*/*M*. *tetraphylla* on *H*. *trifolii* moths captured in Beihuang Island from April to June. These two plants grow in Guangdong, Yunnan and Taiwan provinces in China; thus, the *H*. *trifolii* moths collected on Beihuang Island in the spring probably traveled about 2000 km from southern China. The geographical origin of *H*. *trifolii* in this study is similar to that found for other migratory noctuid moths (e.g., *A*. *ipsilon*, *A*. *segetum* and *M*. *separata*) collected on Beihuang Island (Chang et al., 2018; Liu et al., 2016; Liu, Fu, et al., 2017). Understanding the geographical origin of *H*. *trifolii* can help strengthen the management and control of this pest and secure supplies of major agricultural products (Wu et al., 2022).

We did not find any sex‐related differences in the frequency of pollen attachment, similar to findings for the migratory noctuid moths *A*. *ipsilon*, *A*. *segetum* and *M*. *separata* (Chang et al., 2018; Liu et al., 2016; Liu, Fu, et al., 2017). This result may be due to the fact that both male and female migratory noctuid moths must feed on plants to meet nutritional requirements for the development of the internal reproductive system and for energy for flight, mating, and/or oviposition and other processes (Gilbert, 1972; He et al., 2021; Wäckers et al., 2007; Wu & Guo, 1997; Zhou et al., 2019). Pollen adherence frequency on insects varies obviously among different insects; for example, 68.3% of male *H*. *zea* moths (Hendrix et al., 1987) and 38.4% of female and 65% of male *A*. *ipsilon* carried pollen (Hendrix & Showers, 1992). We found that 11.11% to 37.15% of female *H*. *trifolii* moths and 14.66% to 35.77% of the male moths carried pollen, similar to our findings for migratory *A*. *segetum* and *H*. *armigera* moths captured in Beihuang Island (Chang et al., 2018; Zhou et al., 2019). In general, pollen prevalence on *A*. *segetum*, *H*. *armigera* and *H*. *trifolii* was obviously lower than on *A*. *ipsilon* and *H*. *zea* moths. Diverse elements, involving plant phenology, nectar viscosity, pollen grain characteristics, migratory route, and antennae or mouthpart structure of insects can affect flower visitation patterns and associated variability in the frequency that an insect carries pollen (Krenn, 2010; Liu et al., 2016; Tudor et al., 2004). In addition, moth collection methods may also alter the detection rate of pollen adherence. Migratory noctuid moths captured on Beihuang Island were collected using a searchlight trap, with numerous species and individuals of insects (Guo et al., 2020). A large number of insects are collected in a bag and may be squeezed or rubbed together, which may knock off adherent pollen. However, sex pheromone traps are highly specific and capture fewer insect species than light traps; thus, pollen is less likely to be removed.

The frequency of pollen attachment to field‐collected insects can also be used to infer the relative importance or contribution of the nectar plants (Chang et al., 2018; Jones & Coppedge, 1999). We found notable seasonal differences in the families represented by pollen type on migratory *H*. *trifolii* moths, with more Pinaceae early in the migratory season, Amaranthaceae middle season, and Asteraceae late in the season. This finding was similar to that for *A*. *segetum* (Chang et al., 2018). Plant phenology and species‐specific flowering or pollen‐shedding mechanism can explain seasonal variability in the taxa available as foraging resources for insects and thus represented by adhering pollen. Most plants including Pinaceae bloom in the spring, while Apocynaceae bloom in the summer, and most Asteraceae flower in the autumn. Overall, flowering plants from at least 42 families, including Amaranthaceae, Asteraceae, Fabaceae, Oleaceae, Pinaceae, Polygonaceae, and Rosaceae, were the primary foraging resources for migratory *H*. *trifolii* moths. The area pollinated by *H*. *trifolii* moths may thus extend to all of China during its different migratory seasons.

During their close interactions, plants and insects coevolve. Herbivorous and flower‐visiting insects feed on the roots, stems, leaves, pollens, or nectars of plants, which depend on pollinators for fertilization and cross breeding that may increase genetic diversity and improve quality and yield (Barone, 1998; Bashir et al., 2018; Benjamin & Winfree, 2014; Hendrix & Showers, 1992; Matsuka & Sakai, 2015; Parker, 1981). Generally, flower‐visiting and nectar‐feeding insects can be attracted by floral volatile compounds (Haber et al., 2018; Kessler et al., 2019; Mas et al., 2020). Host plant volatiles (especially floral volatiles) have been proposed as trap baits and a means to monitor and predict populations of moths (Guédot et al., 2008; Tingle & Mitchell, 1992). Our findings indicate that *H*. *trifolii* moths are effective nocturnal pollinators of *A*. *altissima*, *C*. *album*, *F*. *virosa*, *H*. *annuus*, *M*. *azedarach*, *Pinus* spp., *Artemisia* spp., *Cynanchum* spp., *Syringa* spp. and others. The flowers of these plants may produce attractant volatiles, and identifying these volatiles may allow us to develop floral attractants for monitoring and ecofriendly prevention and control of *H*. *trifolii*. On the other hand, *H*. *trifolii* undertakes regular migration across different agricultural areas and could enable genetic exchange among plants across large regions similar to other noctuid pollinators (Chang et al., 2018; He et al., 2018; Hendrix et al., 1987; Liu et al., 2016; Liu, Fu, et al., 2017).

Moth abundance has decreased significantly in recent decades, and their occurrence is likely to be affected by many environmental factors including light pollution and changes in land‐use and climate (Fox et al., 2011, 2014; Macgregor et al., 2015; Péter et al., 2020). The larvae of most lepidopteran insects are agricultural and forestry pests, while the adults (moths or butterflies) are usually pollinators of many plant species and a food source for many organisms (such as birds, bats, fishes, frogs, and spiders) (Devoto et al., 2011; Fox, 2013; Kato & Kawakita, 2017; Liu, Fu, et al., 2017). Therefore, a decrease in moth abundance will also affect the abundance of other organisms in the ecosystem. The ecological functions of agricultural pests need to be continuously explored and assessed. With regard to pollination ecology, the contribution of pollinators other than bees (e.g., beetles, flies, moths, and butterflies) have been little explored although their role in pollination processes is well known (Devoto et al., 2011; Galliot et al., 2017; Lecroy et al., 2013; Matsuka & Sakai, 2015; Weiss, 2001). In the present study, we have provided evidence that *H*. *trifolii* moths are nocturnal pollinators of a diverse range of plant species, but the extent of their role as pollinators in agro‐ecosystems, especially of commercially valuable crops, and as contributors to plant diversity, needs further study.

## 5 CONCLUSIONS

Pollen grain identification is a practical means to study pollination ecology, insect movement, and plant–insect interactions. Here, by identifying plant species using pollen, we gained a better understanding of the interactions between *H*. *trifolii* moths and a wide range of host plants in China. Our work advances the knowledge of the nutrient relationship between a long‐distance migration noctuid insect and its host plants over broad geographical scales, provides valuable and unique information on the nutrition, geographical origin and pollination service of *H*. *trifolii* moths and establishes a basis for targeted control of a global agricultural pest.

## CONFLICT OF INTEREST

The authors declare no conflict of interest.

## AUTHOR CONTRIBUTIONS

**Limei He:** Conceptualization (equal); Data curation (lead); Formal analysis (lead); Investigation (lead); Methodology (equal); Project administration (equal); Resources (equal); Software (lead); Validation (lead); Visualization (lead); Writing – original draft (lead); Writing – review & editing (equal). **Yongqiang Liu:** Data curation (supporting); Formal analysis (supporting); Investigation (supporting); Methodology (supporting); Resources (supporting); Supervision (supporting); Visualization (supporting); Writing – original draft (supporting); Writing – review & editing (supporting). **Jianglong Guo:** Data curation (supporting); Formal analysis (supporting); Investigation (supporting); Resources (supporting); Software (supporting); Writing – original draft (supporting); Writing – review & editing (supporting). **Hong Chang:** Conceptualization (supporting); Formal analysis (supporting); Investigation (supporting); Methodology (supporting); Software (supporting); Writing – original draft (supporting); Writing – review & editing (supporting). **Kongming Wu:** Conceptualization (equal); Data curation (supporting); Formal analysis (lead); Funding acquisition (supporting); Investigation (supporting); Methodology (supporting); Project administration (equal); Resources (supporting); Software (supporting); Supervision (supporting); Validation (supporting); Visualization (supporting); Writing – original draft (supporting); Writing – review & editing (equal).

## Supporting information

Text S1

## Data Availability

The data that support the findings of this study are available in the Supplementary material of this article.
